# Monoclonal antibodies against muscle actin isoforms: epitope identification and analysis of isoform expression by immunoblot and immunostaining in normal and regenerating skeletal muscle

**DOI:** 10.12688/f1000research.8154.2

**Published:** 2016-06-01

**Authors:** Christine Chaponnier, Giulio Gabbiani

**Affiliations:** 1 Department of Pathology-Immunology, Faculty of Medicine, University of Geneva, Geneva, Switzerland

**Keywords:** Actin isoforms, monoclonal antibodies, epitope, muscle repair

## Abstract

Higher vertebrates (mammals and birds) express six different highly conserved actin isoforms that can be classified in three subgroups: 1) sarcomeric actins, α-skeletal (α-SKA) and α-cardiac (α-CAA), 2) smooth muscle actins (SMAs), α-SMA and γ-SMA, and 3) cytoplasmic actins (CYAs), β-CYA and γ-CYA. The variations among isoactins, in each subgroup, are due to 3-4 amino acid differences located in their acetylated N-decapeptide sequence. The first monoclonal antibody (mAb) against an actin isoform (α-SMA) was produced and characterized in our laboratory in 1986 (Skalli *et al*., 1986) . We have further obtained mAbs against the 5 other isoforms. In this report, we focus on the mAbs anti-α-SKA and anti-α-CAA obtained after immunization of mice with the respective acetylated N-terminal decapeptides using the Repetitive Immunizations at Multiple Sites Strategy (RIMMS). In addition to the identification of their epitope by immunoblotting, we describe the expression of the 2 sarcomeric actins in mature skeletal muscle and during muscle repair after micro-lesions. In particular, we analyze the expression of α-CAA, α-SKA and α-SMA by co-immunostaining in a time course frame during the muscle repair process. Our results indicate that a restricted myocyte population expresses α-CAA and suggest a high capacity of self-regeneration in muscle cells. These antibodies may represent a helpful tool for the follow-up of muscle regeneration and pathological changes.

## Introduction

Expression of actin isoforms in skeletal muscle at the mRNA and protein levels have been described since the 1980s using tissue extracts ( Gunning *et al.*, 1983; Hayward & Schwartz, 1986; Hayward *et al.*, 1988; Minty *et al.*, 1982; Ordahl, 1986; Paterson & Eldridge, 1984; Sassoon *et al.*, 1988; Vandekerckhove *et al.*, 1986). These studies have indicated that sarcomeric actins (α-CAA and α-SKA) are expressed in fetal and regenerating skeletal muscle, whereas α-SKA becomes the predominant actin isoform in mature skeletal muscle. Noteworthy, during fetal life, another actin isoform, α-SMA, is highly expressed and precedes the sarcomeric isoforms ( Bochaton-Piallat *et al.*, 1992; Hayward & Schwartz, 1986; McHugh *et al.*, 1991; Woodcock-Mitchell *et al.*, 1988).

Nevertheless, very little is known concerning the precise localization of these isoforms due to the lack of specific α-SKA and α-CAA antibodies. The first mAb against α-CAA has allowed the identification, by immunohistochemistry, of a α-CAA transient expression in human skeletal muscle satellite cells during skeletal regeneration induced by muscle injury, while normal skeletal muscle was negative ( Franke *et al.*, 1996). Ten years later, the same group, in a more extensive study, has further analyzed the expression and localization of α-CAA in normal, regenerating, diseased and neoplastic human muscle tissues ( Moll *et al.*, 2006). In human fetal skeletal muscle, a uniform strong α-CAA staining was observed while in normal adult skeletal muscle, α-CAA was identified only in few thin fibers. α-CAA staining of thin fibers became stronger in human regenerating skeletal muscle after traumatic injury, as well as in Duchenne muscular dystrophy (DMD). Whether the persistence of α-CAA in DMD myofibers is due to a lack of differentiation or to a regenerative process deserves further examination. Interestingly, resting satellite cells in healthy adult muscle lack this isoform, whereas, when satellite cells are activated during the regeneration process, α-CAA is up-regulated. Unfortunately, in these two studies, a comparative α-SKA staining was not performed.

In a more recent study, the switch of α-CAA to α-SKA during the differentiation of skeletal muscle from mouse embryonic stem cells has been examined ( Mizuno *et al.*, 2009). In this *in vitro* system, α-CAA appeared first in myoblasts, with no staining for α-SKA. During cell fusion, α-SKA appeared. When myotubes began to form sarcomeres, α-SKA expression increased while α-CAA began to decrease. Finally, mature skeletal muscle fibers were mainly composed of α-SKA. Although, this *in vitro* system seems to recapitulate the *in vivo* skeletal muscle differentiation at the level of sarcomeric actins switching, the *in vivo* origin of progenitor cells during *in vivo* muscle differentiation/repair remains elusive.

The sequential expression of the two striated actins during: i) heart development (high expression of α-SKA at birth, predominant expression of α-CAA in differentiated cardiac muscle at adult life) and ii) skeletal muscle development (high expression of α-CAA at birth, predominant of α-SKA in differentiated skeletal muscle at adult life) has been known from mRNA studies for decades, but little is known about the distribution and/or the localization of the two α-sarcomeric muscle actins, because of the lack of double immunostainings availability. Nevertheless, our laboratory, in collaboration with others, has studied α-SKA expression and distribution, in particular in developing and pathological hearts, using affinity polyclonal antibodies ( Clément *et al.*, 1999), again without double immunostaining.

As mentioned above, α-CAA distribution has been studied during muscle development and repair ( Moll *et al.*, 2006), but a comparative study with α-SKA was still missing. Our mAbs were raised using the acetylated N-terminus decapeptide of each isoform (see Table 1). The two different mAb subtypes (anti-α-SKA, IgG2b and anti-α-CAA, IgG1), allowed a clear analysis of the expression and distribution of the two actin isoforms in mature skeletal muscle and during regeneration after micro-lesions by means of highly specific anti- mouse subtype secondary antibodies.

**Table 1.** List of monoclonal antibodies against the 6-actin isoforms raised in our laboratory. The epitope recognized by each antibody is enlightened in bold.

Actin isoform	Immunogen	MAb name	Clone	Subtype	Original Reference	Distributors	Catalog Number
α-SMA	**AcEEED**STALVC	Anti-α-SM1	1A4	IgG2a	Skalli *et al.*, 1986	Abcam AbD Serotec Cell Marque Corporation Dako eBioscience EMD Millipore Genemed Biotechnologies Nordic-MUbio R§D Systems Santa Cruz Biotechnology Sigma-Aldrich Spring Bioscience Zeta Corporation	ab-7817 MCA5781GA 202M M0851 14–9760 113200 61–0001 MUB0107S MAB1420 sc-32251 A5228 E14344 Z2066
γ-SMA	**AcEEETTALV**C	Anti-γ-SMA	20D2	IgG1	Arnoldi *et al.*, 2013	NA	NA
β-CYA	**AcDDDI**AALVC	Anti-β-CYA	4C2	IgG1	Dugina *et al.*, 2009	AbD Serotec EMD Millipore Nordic-MUbio	MCA5775GA MABT825 MUB0110S
γ-CYA	**AcEEEIAAL**VC	Anti-γ-CYA	2A3	IgG2b	Dugina *et al.*, 2009	AbD Serotec EMD Millipore Nordic-MUbio	MCA5776GA MABT824 MUB0111S
α-SKA and α-CAA Sarcomeric actins	Rabbit skeletal actin	Anti-α-SR1	5C5	IgM	Skalli *et al.*, 1988	Dako Santa Cruz Biotechnology Sigma-Aldrich Spring Bioscience	M0874 sc-58670 A 2172 E16654
α-SKA	**AcDEDETTA**LVC	Anti-α-SKA	3B3	IgG1	Driesen *et al.*, 2009	Nordic-MUbio	MUB0108S
α-SKA	**AcDEDETTA**LVC	Anti-α-SKA	10D2	IgG2a	Driesen *et al.*, 2009	NA	NA
α-CAA	**AcDDEET**TALVC	Anti-α-CAA	22D3	IgG1	Driesen *et al.*, 2009	EMD Millipore Nordic-MUbio	MABT823 MUB0109S

## Materials and methods

### Reagents details

Details of all reagents with reference to the immunoblot and immunostaining procedure can be found in Table 2 and Table 3. Crucial are the conditions of fixation and permeabilization for relevant immunostaining. With cells in culture as well as with tissues, we have tested a large number of conditions such as MeOH, EtOH, PFA-TX100. By far, the use of PFA, followed by MeOH, as described in Table 3 and previously defined ( Dugina *et al.*, 2009), allowed the best detection of every actin isoform, likely because of availability of the actin molecule N-terminus.

**Table 2.** Western blot protocol for the identification of the epitopes recognized by anti-α-SKA (clone 10D2) and anti-α-CAA (clone 22D3).

Protocol steps	Reagent	Concentration/ dilution	Time
Migration of Purified α-SKA and α-CAA on SDS-PAGE		1μg/lane	overnight
• Transfer on Nitrocellulose membranes. • Membranes cut in strips for Ab-peptides incubation.			2 h
Preincubation of antibodies with peptides	MAb: • anti-α-SKA (10D2) • anti-α-CAA (22D3) For details, see Table 1 Blocking Peptides (see Figure 1)	1:5000 1:5000 100 μg/ml	1 h
Membranes pretreatment	5% dried skimmed milk in TBS	5%	1 h
Membranes Incubation with antibody-peptides	In 0.1% TX-100/TBS		2 h
Membranes Incubation with 2 ^nd^ antibody	Goat Anti-Mouse IgG (H+L) HRP Conjugate (BioRad) #1706516 In 0.1% TX-100/TBS	1:10’000	1 h
HRP activity development	ECL protocol according to (RPN 2109; GE Healthcare),		5–60 sec

**Table 3.** Details of immunofluorescence staining protocol.

Protocol steps	Reagent	Concentration /dilution	Time	Catalogue Number
• Cryopreserved Rat muscles • Cryostat sections (3μm)				
Fixation	• Paraformaldehyde • Methanol	1% 100%	30 min at RT 5 min at –20°C	
Staining	Primary Abs: MAbs: • anti-α-SKA (10D2) • anti-α-CAA (22D3) • anti-α-SMA (1A4) For details, see Table 1 • anti-vimentin (clone V9) *Immunogen:* Purified vimentin from porcine eye lens *Specificity:* the antibody cross-reacts with vimentin in man, cow, dog, hamster, horse, rhesus and African green monkey, rabbit, rat and rat kangaroo. No cross-reaction with mouse vimentin could be demonstrated, and results on chicken specimens are contradictory. Rabbit polyclonal Ab: • anti-α-CAA ( Clément *et al.*, 2003)	1:50 1:50 1:50 1:300 1:100	2 h	Home made Home made Home made Dako #M 0725 Home made
	Secondary Abs (Jackson Immmunoresearch): • AffiniPure Goat Anti-Mouse IgG1, Alexa 488 • AffiniPure Goat Anti-Mouse IgG1, Alexa 594 • AffiniPure Goat Anti-Mouse IgG2a, Alexa 488 • AffiniPure Goat Anti-Mouse IgG2a, Alexa 594 • AffiniPure Goat Anti-Rabbit-Alexa 594	1:200 1:200 1:200 1:200 1:100	1 h	115-545-205 115-585-205 115-545-206 115-585-206 111-585-144

### Animals

All animal experiments (production of antibody in mice, rat wounds, tissue samplings) were approved by and performed in accordance with the cantonal and federal veterinary authorities.

### Tissue details

Tibialis muscle specimens from female Wistar rats older than 10 weeks were rapidly embedded in OCT compound (Tissue-Tek), snap-frozen in a beaker containing precooled liquid isopentane, immerged in liquid nitrogen, and stored at -80°C. For muscle repair studies, rat tibialis muscles were injured with a liquid nitrogen cooled needle, using a well-set simple rat model ( Rocheteau *et al.*, 2012). Muscles were taken, OCT embedded and frozen at different days after injury.

### Antibody production and details

MAbs against α-SKA and α-CAA were prepared following the Repetitive Immunizations Multiple Sites (RIMMS) strategy ( Kilpatrick *et al.*, 1997). This strategy uses lymphocytes from regional draining lymph nodes and an immunization schedule significantly shorter than conventional techniques: two weeks instead of several months. Along with the use of less antigen, this approach allows to obtain hybridomas in a month.

Mice were immunized with the acetylated N-terminal decapeptide of α-SKA (Ac-DEDETTALVC-COOH) or α-CAA (Ac-DDEETTALVC-COOH) conjugated with keyhole limpet haemocyanin through the cysteine peptide C-terminus (KLH, Imject Maleimide Activated carrier proteins, Pierce) according to the instructions of the manufacturer. Briefly, over a period of 10 days, 5 injections of 5 μg of protein (α-SKA or α-CAA peptide x KLH) emulsified in complete Freund’s adjuvant (first injection) or with incomplete Freund’s adjuvant (for the remaining injections) and RIBI adjuvants (Sigma-Aldrich) were given at six subcutaneous sites proximal to draining peripheral lymph nodes (PLNs) in three anesthetized six-week-old female BALB/c mice (200μl/mice). Two days after the final boosts, animals were sacrificed. PLNs were harvested from popliteal, superficial inguinal, axillary, and brachial lymph nodes and dissociated. Up to 1 × 10 ^8^ lymphocytes per 3 mice, were used for fusions with 2.5 × 10 ^7^ with NSO myeloma cells using 50% polyethylene glycol (PEG 1500, Sigma-Aldrich). Fused cells were plated in 24-well tissue culture plates (4000 cells/well). Hybridomas were grown in DMEM+ pyruvate, 5% FCS, 10% NCTC 109 (Sigma-Aldrich), 1× MEM Non-Essential Amino Acids (Gibco), 1% Hybridoma Fusion and Cloning Supplement (HFCS, Roche), penicillin/streptomycin, and 1× selective media hypoxanthine/aminopterin/thymidine (HAT). Supernatants were harvested after 2 weeks and screened for antibody specificity by ELISA and immunofluorescence staining (see below). Hybridomas from wells of interest were distributed into 96-well plates (1-2-5-10 cells/well) for the first limited dilution using 1× hypoxanthine and thymidine (HT) in place of HAT. After about 10 days of culture, wells with single clones were identified by microscope and their supernatants were harvested for screening. Positive clones were re-plated using another limited dilution for a further 10 days in DMEM containing 10% FCS and antibiotics. Clones secreting the specific mAb were expanded and frozen. Hybridoma were cultivated at post confluence in the same medium for mAb production. Sodium azide (0.1%) was added to the supernatants for storage. See Table 2 and Table 3 for working dilution.

Supernatants of hybridoma cells secreting anti-α-SKA or anti-α-CAA were screened by: i) triple ELISA, using 96 well plates coated with α-SMA, α-SKA and α-CAA BSA-conjugated peptides, using Maleimide Activated BSA (Pierce) according to the instructions of the manufacturer; ii) Western blotting using platelets, heart, aorta, skeletal muscle, gizzard extracts ( Driesen *et al.*, 2009); iii) immunofluorescence using rat lip, skeletal and heart sections. Selected hybridomas were cloned twice by limited dilution, as described above, and the final mAB characterization was performed by immunoblotting after transfer of one-dimensional gels containing tissue and cell extracts and finally by blocking assays ( Chaponnier *et al.*, 1995) using the N-terminal peptides of α-SKA and α-CAA.

### Electrophoresis and immunoblot analysis

**Figure 1.**
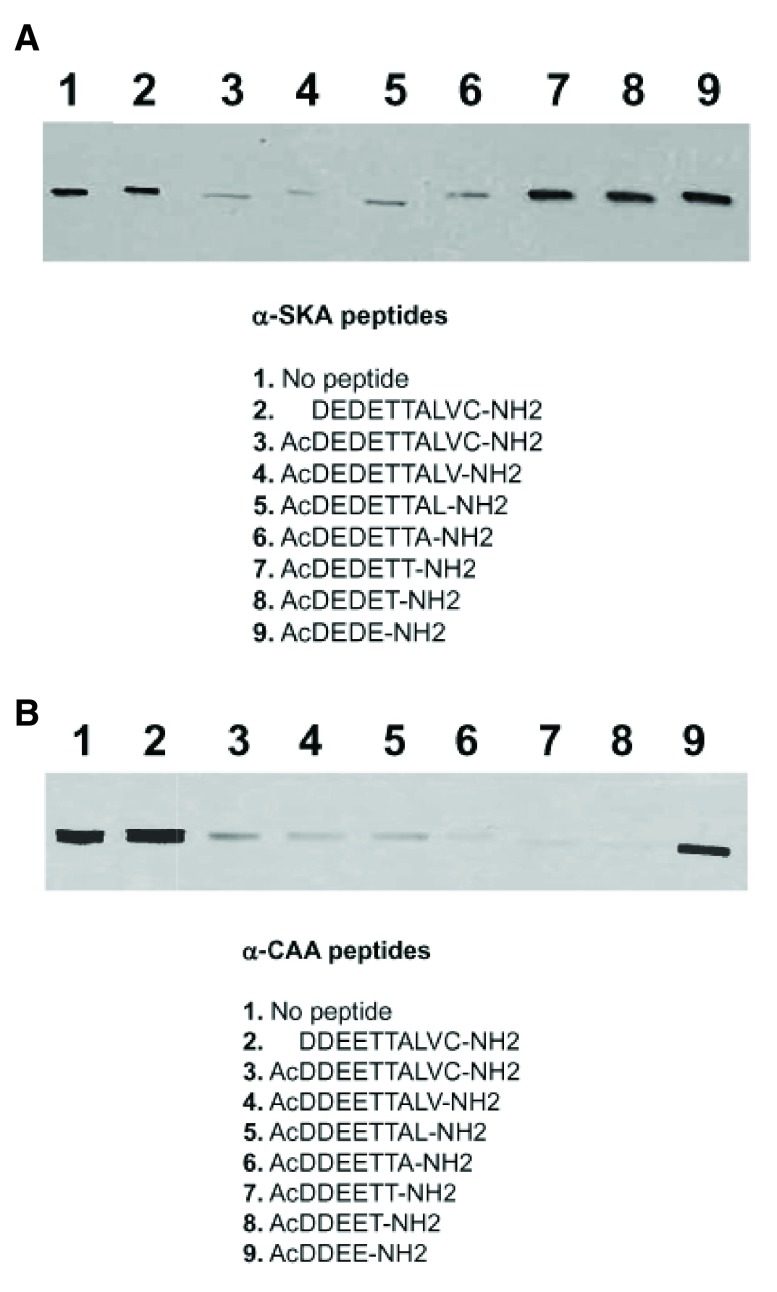
Identification, by immunoblots, of the epitope recognized by anti-α-SKA ( **A**) and anti-α-CAA ( **B**). Purified α-SKA ( **A**) or α-CAA ( **B**) was run on 10% SDS-PAGE and transferred on nitrocellulose membrane. Membrane strips were incubated with the mAb anti-α-SKA ( **A**) or anti-α-CAA ( **B**) alone (control, lane 1) or mixed with the listed peptides (2–9). The epitope recognized by anti-α-SKA ( **A**) includes the acetyl group and the first 7 amino acids of the α-SKA sequence. The epitope recognized by anti-α-CAA ( **B**) includes the acetyl group and the first 5 amino acids of the α-CAA sequence.

Purified α-SKA ( Spudich & Watt, 1971) and α-CAA ( Zot & Potter, 1981) were run on 10% SDS-PAGE ( Laemmli, 1970) and electroblotted to nitrocellulose according to Towbin *et al.* ( Towbin *et al.*, 1979). After preincubation with the respective different length peptides (listed in Figure 1), the antibodies, diluted in Tris-buffered saline (TBS) solution containing 3% BSA and 0.1% Triton X-100, were incubated on membranes strips for two hours at room temperature. After 3 washes with TBS, a second incubation was performed with peroxidase-conjugated affinity purified goat anti-rabbit IgG (Biorad) at a dilution of 1:10,000 in TBS containing 0.1% BSA and 0.1% Triton X-100. Peroxidase activity was developed using the ECL Western blotting system (GE Healthcare), exposed to AX Konica Minolta films for 5–60 sec, and processed with Curix-60 developing machine (Agfa). Blots were scanned and quantified using densitometric analysis ImageJ v1.49 software (NIH, http://rsb.info.nih.gov/ij/).

### Immunofluorescence microscopy

Cryopreserved tissues were sliced (3 μm) with a cryostat microtome (Microm). Sections were positionned on glass slides, fixed with 1% PFA for 30 min at room temperature, followed by three washes with PBS and a 3 min treatment with methanol at -20°C. After three 5 min washes with PBS at RT, tissue sections on glass slides were incubated with primary (2h) and secondary antibodies (1h) at appropriate dilutions (see Table 3). Normal rat serum (1:50) was used to block non-specific sites, and DAPI for nuclear staining. After washing in PBS, sections on slides were mounted in polyvinyl alcohol (PVA: 50 mM Tris-phosphate pH 9.0, 0.1% chlorobutanol, 20% polyvinyl alcohol, 0.5‰ phenol red, 20% glycerol) ( Lennette, 1978). Images were acquired using a Zeiss Axiophot microscope (Carl Zeiss), equipped with plan apochromatic 10x, 20x, or 40x objectives and a high sensibility color camera (Axiocam, Zeiss).

After a careful selection of highly specific secondary antibodies against mouse subtypes, testing a large panel of these antibodies, we selected those commercialized by Jackson Immunoresearch and Southern Biotechnology.

## Results

Raw data for Figure 1a,b Click here for additional data file. Copyright: © 2016 Chaponnier C and Gabbiani G 2016 Data associated with the article are available under the terms of the Creative Commons Zero "No rights reserved" data waiver (CC0 1.0 Public domain dedication).

### Identification of the epitopes recognized by the actin isoform antibodies

Acetylated N-terminal peptide of different length (4–10 amino acids) and non-acetylated N-terminal decapeptide of α-SKA and α-CAA were tested for their blocking ability of the respective mAb ( Figure 1). We identified the epitope of α-SKA (AcDEDETTA) and of α-CAA (AcDDEET). The epitope of the other actin isoforms were previously identified and are listed in Table 1 (in bold letters). Noteworthy, the acetyl group is a critical element of the epitope of each isoform.

### Comparison of distribution of α-SKA and α-CAA, in mature skeletal muscle

**Figure 2.**
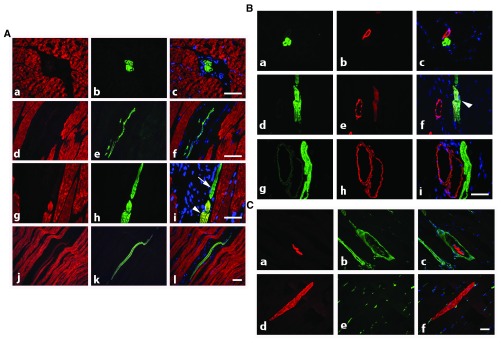
Rat adult skeletal muscle co-stained with isoform specific antibodies. **A**) Co-staining with anti-α-SKA (red) and anti-α-CAA (green) allows the detection of isolated thin α-CAA positive fibers in interstitial connective tissue (transversal sections, a–c: longitudinal section, d–f) and of regenerating muscle fibers expressing both isoforms (longitudinal section, g–l, arrowhead), or only α-CAA (arrow). Merged images are shown on right column. Bars = 50 μm. **B**) Co-staining with anti-α-CAA (green) and anti-α-SMA (red) shows that α-CAA positive spindle cells are in close connection with α-SMA positive vessel (a–c), that early muscle fiber self-regeneration is characterized by co-expression of both isoform (d–f, arrowhead), although more advanced regenerated fibers are only α-CAA positive (g–i). Merged images are shown on right column. Bar = 50 μm. **C**) Co-staining with anti-α-CAA (red) and anti-vimentin (green) allows the detection of α-CAA positive muscle spindle cells surrounded by a capsule containing vimentin positive cells (transversal section, a–c) and of regenerating α-CAA positive fibers in contact with vimentin positive cells (longitudinal section, d–f). Merged images are shown on right column. Bar = 50 μm.

The normal myonuclear turnover in rodents is estimated at 1–2% per week ( Schmalbruch & Lewis, 2000). As a first investigation, we have compared the distribution of α-CAA with α-SKA, α-SMA and vimentin on cryostat sections of adult rat tibialis muscle. Although a minority of myocytes expressed α-CAA, we have observed two types of α-CAA positive cells in skeletal fibers: one was mainly located in interstitial connective tissue ( Figure 2A, a–c, d–f) and the other was localized in the muscle mass ( Figure 2A, g–i, arrowhead). The first type corresponded most likely the “muscle spindles”, described by Moll *et al.* ( Moll *et al.*, 2006), as “modified muscle fibers of neuromuscular spindles, believed to act as sensors of muscle tension”. The “spindle” cells expressed exclusively α-CAA, whereas the “classical muscle fibers”, in addition to a high expression of α-CAA, displayed α-SKA at various levels ( Figure 2A, g–i, j–l), probably according to the state of differentiation during self-regeneration of myocytes.

It is well known that during skeletal muscle development, α-SMA is the first muscle actin to be expressed in myocytes during fetal life. Therefore, we also investigated the expression of α-SMA during muscle self-regeneration. We observed that a few regenerating α-CAA positive fibers displayed α-SMA ( Figure 2B, d–f).

As vimentin is expressed in the capsule of muscle spindle cells ( Cizková *et al.*, 2009), we investigated further whether the α-CAA positive fibers located in interstitial spaces could be identified as component of muscle spindles. After co-staining of muscle sections for α-CAA and vimentin, we confirmed that a capsule containing vimentin-positive cells surrounded isolated α-CAA positive spindle fibers ( Figure 2C, a–c). Noteworthy, regenerating fibers displayed contacts with vimentin positive cells ( Figure 2C, d–f).

### Distribution of α-SMA, α-SKA and α-CAA, in mature skeletal muscle during the repair process

**Figure 3.**
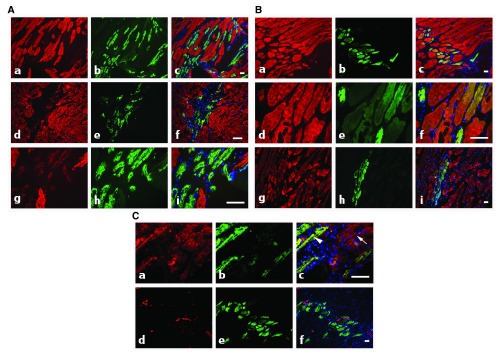
Regenerating rat adult skeletal muscle co-stained with isoform specific antibodies. **A**) At 4d post-injury, co-staining with anti-α-SKA (red) and anti-α-CAA (green) shows the presence of α-CAA positive fibers in the injured muscle area (a–i). At this stage of regeneration, only a few fibers co-express both isoforms (d–i), with a low α-SKA level (g–i). Merged images are shown on right column. Bars = 50 μm. **B**) At 5d–9d post-injury, co-staining with anti-α-SKA (red) and anti-α-CAA (green) shows that after 5d, most fibers co-express both isoforms (a–f), whereas at 9d (g–i), α-SKA positive fibers become predominant. Merged images are shown on right column. Bars = 50 μm. **C**) At 4d–5d post-injury, co-staining with anti-α-SMA (red) and anti-α-CAA (green) shows that during the healing process, only a few fibers co-express both isoforms after 4d (a–c, arrowhead), whereas after 5d, only α-CAA positive fibers are detected (d–f). After 4d, myofibroblasts might participate to the repair process and are detected by using the anti-α- SMA mAb (a–c, arrow). Merged images are shown on right column. Bars = 50 μm.

The use of specific mAbs against actin isoforms allows the tracking and follow-up of myocytes regeneration during the muscle repair process. In particular, complete and fast repair can be observed after muscle micro-lesions obtained after light injury induced by nitrogen-cooled needle application. At early stage (4 day post-injury), an important population of α-CAA positive myofibers was observed ( Figure 3A) at injury sites. These cells, being α-SKA negative or marginally positive, are probably in an early stage of differentiation. Few of them co-expressed α-SMA ( Figure 3C, a–c, arrowhead). Only rarely, α-SMA positive myofibroblasts, the hallmark of fibrotic process ( Tomasek *et al.*, 2002) were detected ( Figure 3C, a–c, arrow). At 5d post-injury, fibers started to express more importantly α-SKA in addition to α-CAA in the regenerating location ( Figure 3B, a–f). In our model, muscle regeneration was very rapid, as expression of α-SMA was not any longer detectable 5d after injury ( Figure 3C, d–f). At 9d post-injury, co-expression of α-SKA and α-CAA became rare ( Figure 3B, g–i). These results suggest that skeletal muscle has a high capacity of regeneration after micro-injury and that actin isoform specific mAbs represent an important tool for muscle regeneration tracking.

## Conclusion

In conclusion, α-CAA, in conjunction with the expression of α-SMA and α-SKA, appears to represent a valuable marker for the identification of myofibers regeneration in skeletal muscle and for the analysis of the degree of fiber differentiation. For this purpose, it is important to use well-characterized and specific antibodies. Furthermore, high quality anti-mouse subtype secondary antibodies allow double immunostaining necessary for this type of investigation.

## Data availability

The data referenced by this article are under copyright with the following copyright statement: Copyright: © 2016 Chaponnier C and Gabbiani G

Data associated with the article are available under the terms of the Creative Commons Zero "No rights reserved" data waiver (CC0 1.0 Public domain dedication). 

*F1000Researc*h: Dataset 1. Raw data for Figures 1a, b., 10.5256/f1000research.8154.d117164 ( Chaponnier & Gabbiani, 2016).

## Figures and Tables

**Supplemental Figure 1.**
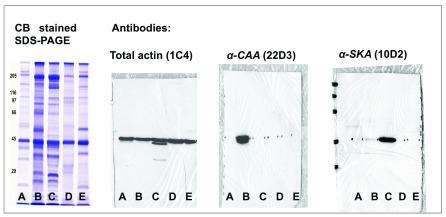
Immunodetection of α-CAA and α-SKA in total tissue extracts by Western blots. Coomassie Blue (CB) staining of bovine aorta (A), rat heart (B), rat skeletal muscle (C), human platelets (D), and chicken gizzard (E) total extracts. Immunodetection of total actin, α-CAA and α-SKA with specific mAbs in total extracts of the same tissues.

**Supplemental Figure 2.**
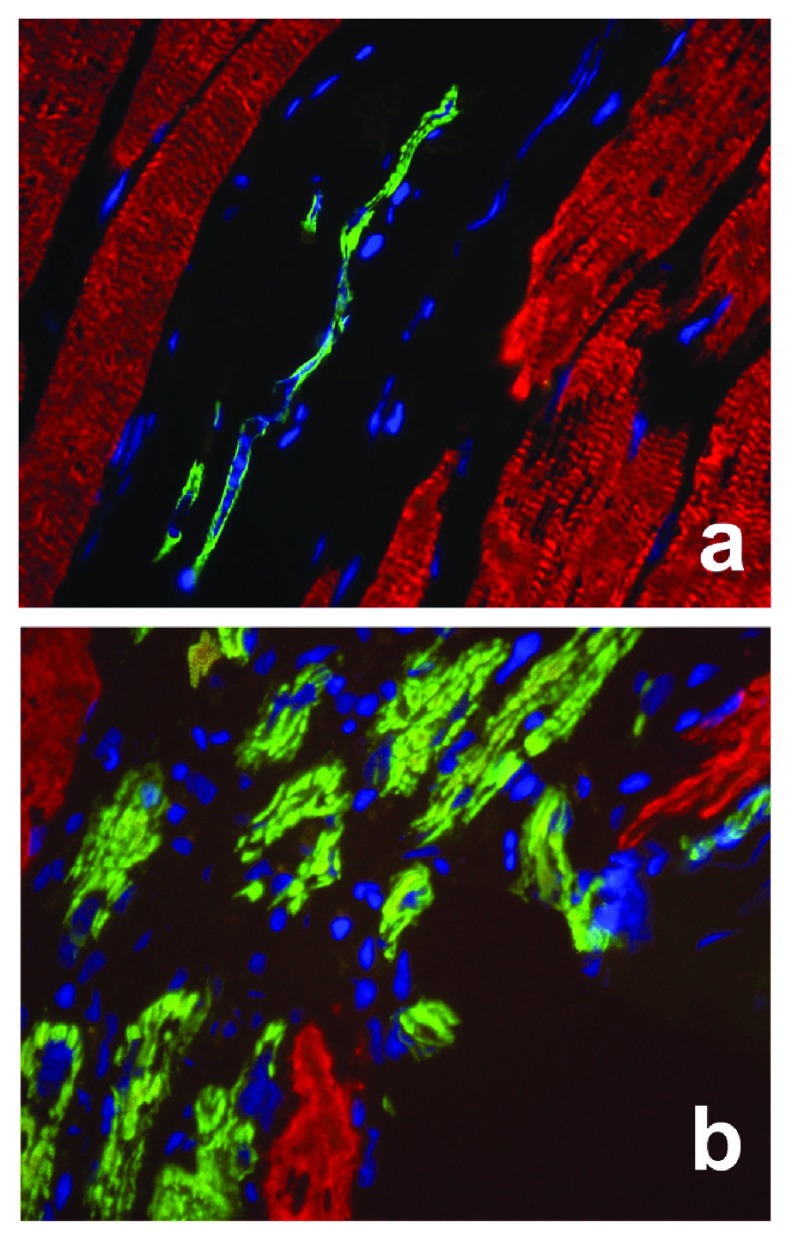
Enlargement of Figure 2Af ( **a**) and Figure 3Ai ( **b**), showing that, either in normal adult muscle ( **a**), or at 4d post-injury during the healing process ( **b**), regenerating αCAA (green) positive myofibers with centrally located nuclei (blue) are detectable. Differentiated muscles express αSKA (red) with peripheral located nuclei (blue) in both situations.
