# Supplementary material for: Monoclonal antibodies against muscle actin isoforms: epitope identification and analysis of isoform expression by immunoblot and immunostaining in normal and regenerating skeletal muscle
Source: F1000Res. 2016 Jun 1;5:416. Originally published 2016 Mar 30. [Version 2] doi: 10.12688/f1000research.8154.2 (PMC4893938; doi:10.12688/f1000research.8154.2)
Supplement: Raw data for Figure 1a,b [file f1000research-5-9468-s0000.tgz › 96994325-2021-41e5-a241-4f5094a42009_Rawdata.docx]

**Figure 1A_raw data**

Table of α-SKA peptides

| Peptide | Position | Sequence | Synthesis |
| --- | --- | --- | --- |
| No peptide | 1 and 12 | ------------------------- |  |
| α-SKA | 2 and 13 | Ac- DEDETTALVC -NH2 | Home made |
|  | 3 and 14 | Ac- DEDETTALVC -NH2 | Affinity Research |
|  | 4 and 15 | H- DEDETTALVC -NH2 | Mimotope |
|  | 5 and 16 | Ac- DEDETTALVC -NH2 | Mimotope |
|  | 6 and 17 | Ac- DEDETTALV -NH2 | Mimotope |
|  | 7 and 18 | Ac- DEDETTAL -NH2 | Mimotope |
|  | 8 and 19 | Ac- DEDETTA -NH2 | Mimotope |
|  | 9 and 20 | Ac- DEDETT -NH2 | Mimotope |
|  | 10 and 21 | Ac- DEDET -NH2 | Mimotope |
|  | 11 and 22 | Ac- DEDE -NH2 | Mimotope |

**Blocking peptide effect on α-SKA mAbs (clone 3B3 and clone 10D2)**

- Purified α-SKA run on SDS-PAGE and transferred on Nitrocellulose membrane
- Membrane cut in strips
- mAb incubated with SKA peptides described in table above for 1h
- Incubation 1^st^ Ab for 2h
- Incubation 2^nd^ Ab for 1h
- HRP development

For details, see Table 2

**Figure 1B_raw data**

Table of α-CAA peptides

| Peptide | Position | Sequence | Synthesis |
| --- | --- | --- | --- |
| No peptide | 1 | ------------------------- |  |
| α-CAA | 2 | Ac- DDEETTALVC -NH2 | Affinity Research |
|  | 3 | H - DDEETTALVC -NH2 | Mimotope |
|  | 4 | Ac- DDEETTALVC -NH2 | Mimotope |
|  | 5 | Ac- DDEETTALV -NH2 | Mimotope |
|  | 6 | Ac- DDEETTAL -NH2 | Mimotope |
|  | 7 | Ac- DDEETTA -NH2 | Mimotope |
|  | 8 | Ac- DDEETT -NH2 | Mimotope |
|  | 9 | Ac- DDEET -NH2 | Mimotope |
|  | 10 | Ac- DDEE -NH2 | Mimotope |

**Blocking peptide effect on α-CAA mAbs (clone 22D3)**

- Purified α-CAA run on SDS-PAGE and transferred on Nitrocellulose membrane
- Membrane cut in strips
- mAb incubated with SKA peptides described in table above for 1h
- Incubation 1^st^ Ab for 2h
- Incubation 2^nd^ Ab for 1h
- HRP development

For details, see Table 2
