# Supplementary material for: Monoclonal antibodies against muscle actin isoforms: epitope identification and analysis of isoform expression by immunoblot and immunostaining in normal and regenerating skeletal muscle
Source: F1000Res. 2016 Jun 1;5:416. Originally published 2016 Mar 30. [Version 2] doi: 10.12688/f1000research.8154.2 (PMC4893938; doi:10.12688/f1000research.8154.2)
Supplement: Peer review history

# Peer review: Referee response for version 2

**DOI:** 10.5256/f1000research.9468.r14123

**Published:** 2016-06-02

**Authors:** Naohiro Hashimoto

**Affiliations:** 1 Department of Regenerative Medicine, National Institute for Longevity Sciences, National Center for Geriatrics and Gerontology, Ōbu, Japan

I understand the purpose of this report is the validation of the actin isoforms antibodies. Muscle biologists would like to hear answers for questions derived from this study. 

 For example:

1. What is a role of  myofibroblasts appear in regenerating muscles and disease muscles?
2. Does each actin isoform play a distinct role during  muscle regeneration?

Particularly, a role of aCAA in newly formed myofibers is interesting.

 I expect the panel of antibodies against actin isoforms will help future studies to reveal distinct role(s) of actin isoforms in muscle regeneration.

I have read this submission. I believe that I have an appropriate level of expertise to confirm that it is of an acceptable scientific standard.

# Peer review: Referee response for version 1

**DOI:** 10.5256/f1000research.8771.r13217

**Published:** 2016-04-12

**Authors:** Shoichiro Ono

**Affiliations:** 1 Department of Pathology and Department of Cell Biology, Emory University, Atlanta, GA, USA

Drs. Chaponnier and Gabbiani report production and validation of monoclonal antibodies that specifically recognize alpha-skeletal muscle actin (aSKA) or alpha-cardiac alpha actin (aCAA), which are different only in a few amino acids at the N-terminus. The new antibody against aSKA (10D2) is IgG2a, while anti-aCAA (22D3) is IgG1, which allows researchers to perform double staining in immunohistochemistry to distinguish these closely related actin isoforms. The validation in immunohistochemistry using rat tissues clearly demonstrates specific labeling of different subsets of cells by the isoform-specific antibodies. These antibodies should be outstanding resources to characterize actin isoform expression and subcellular localization in muscle development, regeneration, and diseases, as well as to investigate functions of actin isoforms. I have a few suggestions for a revision mostly for clarification purposes.

1. In abstract and introduction, “higher vertebrates” should be more clearly defined. Classification of six actin isoforms in 3 subgroups applies to mammals and birds. It should be useful for researchers who use non-mammalian or non-avian species to state whether this classification applies to other vertebrates. A recent analysis by Gunning *et al*. ^1^ indicates that zebrafish has 10 muscle actins, suggesting some gene duplications and isoform divergence in fish.
2. Page 3, left column, 3 ^rd^ paragraph, “sarcomers” should be “sarcomeres”.
3. In Materials and Methods “Antibody production and details”, this section should also include description of how the monoclonal antibodies were prepared after the clonal selection has been completed, which should include whether the antibodies were finally prepared in culture media with or without serum or in ascites, and whether protein concentrations were determined.
4. Also In Materials and Methods “Antibody production and details”, the selection process includes screening using Western blotting using extracts from several different tissues. To improve transparency of the validation process, representative images of the Western blots preferably showing a wide molecular weight range should be included to demonstrate specificity.

I have read this submission. I believe that I have an appropriate level of expertise to confirm that it is of an acceptable scientific standard.

## References

[1] GunningPWGhoshdastiderUWhitakerSPoppDRobinsonRC: The evolution of compositionally and functionally distinct actin filaments. *J Cell Sci*.2015;128(11) : 10.1242/jcs.165563 2009-19 10.1242/jcs.165563 25788699

# Response

**Published:** 2016-05-18

**Authors:** Christine Chaponnier

**Affiliations:** University of Geneva, Switzerland

Your careful review and suggestions for improving the report are highly appreciated. Please find below our answers to your questions.

a) We have modified the abstract accordingly.
  b) Concerning the amino acid sequences in zebrafish, the situation is very confusing. Search in databases gives the following results:
  Actin, alpha 2, smooth muscle, aorta
  Danio rerio (Zebrafish) (Brachydanio rerio)
  Q6DHS1-1
  Ac-DDEESTALVC
  Actin, alpha cardiac muscle 1 [Danio rerio]
  Q6IQR3-1
  Ac-DDDETTALVC
  Actin, alpha 1, skeletal muscle
  Danio rerio (Zebrafish) (Brachydanio rerio)
  Q9I8V1-1
  Ac-DDEETTALVC
  According to the available sequence (although not reviewed), the N-terminus sequence of alpha 1, skeletal muscle in Zebrafish is identical to the alpha cardiac actin in mammals and birds!!! Furthermore, the N-terminus of alpha 2, smooth muscle, aorta and of alpha cardiac muscle are different compared to the α-SMA and α-CAA human and bird sequence.
sarcomers changed for sarcomeres.
Additional informations have been included in the "Material and Methods" section.
  Yes the final antibody is obtained and used in “hybridoma supernatant” in the culture media with FCS for two reasons:
  a) Ascites production is not allowed in Switzerland for years.
  b) Hybridoma supernatants production is anyway privileged, as ascites are very often contaminated with unrelated antibodies with production time.
  c) Protein concentration was not determined precisely (in general, between 0.1-10 μg/ml). However, the dilution to be used is indicated in the report.
We provide a new figure (Supplemental figure 1), with raw data of Western blots on different tissue extracts, showing the specificity of the antibodies.

# Peer review: Referee response for version 1

**DOI:** 10.5256/f1000research.8771.r13310

**Published:** 2016-04-11

**Authors:** Omar Skalli

**Affiliations:** 1 Department of Biological Sciences, Integrated Microscopy Center(IMC), University of Memphis, Memphis, TN, USA

The paper by Chaponnier and Gabbiani reports the production of two monoclonal antibodies, one specific for skeletal actin and the other for cardiac actin. Being able to obtain these antibodies is a momentous accomplishment given that these two actin isoforms differ from each other by only a few amino acids. The authors rigorously demonstrate the specificity of each antibodies for their respective isoform by Western blotting and competitive assay with the immunogen. Because the antibodies are from different subtypes, they can be used by double immunofluorescence to investigate the regulation of skeletal and cardiac actin in different conditions. The authors provide stunningly beautiful immunofluorescence images that demonstrate the utility of their antibodies to pinpoint different cellular populations during skeletal muscle repair. It is to be expected that these antibodies will be extremely useful to studies questions related to cardiac and skeletal muscle physiology and pathology.

I have read this submission. I believe that I have an appropriate level of expertise to confirm that it is of an acceptable scientific standard.

# Response

**Published:** 2016-05-18

**Authors:** Christine Chaponnier

**Affiliations:** University of Geneva, Switzerland

Your nice review and perfect understanding concerning the objectives of this report are highly appreciated.

# Peer review: Referee response for version 1

**DOI:** 10.5256/f1000research.8771.r13281

**Published:** 2016-04-08

**Authors:** Naohiro Hashimoto

**Affiliations:** 1 Department of Regenerative Medicine, National Institute for Longevity Sciences, National Center for Geriatrics and Gerontology, Ōbu, Japan

Higher vertebrates express six different highly conserved actin isoforms.  The authors determined the precise localization of alpha-skeletal actin (aSKA) and alpha-cardiac actin (aCAA) in normal and regenerating rat skeletal muscles. The distinct distribution of the two alpha actin isoforms seems very interesting. However, the authors should describe the results of their immunofluorescent analysis on skeletal muscle more precisely and carefully. 

1. The authors often used terms “renewing muscle (fibers)” and “regenerating fibers”.  However, there is no definition of them. The authors should show the presence of centrally located nuclei in those myofibers because those are found in newly formed myofibers.
2. The authors identified myofibroblasts in regenerating skeletal muscles as the alpha-SMA (aSMA) positive mononuclear cells. However, myoblasts also express aSMA.  Thus, the authors should determine the absence of myogenic lineage markers such as MyoD in those cells.
3. Intracellular localization of alpha actin isoforms should be shown in pictures with higher magnification in addition to the present Figure 2 and 3. Those data will provide important information suggesting different function of those actin isoforms.
4. The term “self-renewal” is used incorrectly. For examples, “self-renewal in muscle cells” and “during muscle self-renewal” are difficult to understand.  I wonder whether “muscle cells” mean muscle satellite cells, myoblasts (muscle progenitor cells), or myofibers.°  “Muscle  self-renewal” means muscle regeneration?

I have read this submission. I believe that I have an appropriate level of expertise to confirm that it is of an acceptable scientific standard, however I have significant reservations, as outlined above.

# Response

**Published:** 2016-05-18

**Authors:** Christine Chaponnier

**Affiliations:** University of Geneva, Switzerland

Your careful review and suggestions for improving the report are highly appreciated. Please find below our answers to your questions.

  

a) We have modified the text according to the suggestion using in general the term regeneration and not renewal.
  b) Enlargement of Figure 2Af and 3Ai showing αCAA positive myofibers with centrally located nuclei during muscle regeneration. (See supplemental Figure 2)
a) The injury model used in this study is in general not accompanied by fibrotic events during the healing process. Only, in rare exceptions, as mentioned in the text, myofibroblasts were detected. We have a long experience in the “myofibroblast history”, and according to the α-SMA positivity and the cell shape, we believe that this area is composed of myofibroblasts.
  b) We agree that the absence of MyoD would validate our assumption. However, the purpose of this report is the validation of the actin isoforms antibodies and not a extensive study on muscle regeneration. We hope that muscle experts will take advantage of these tools for more advanced studies.
As indicated above, the purpose of this report is the validation of the actin isoforms antibodies. The intracellular localization of α-SMA, sometimes in striations, will not give clear information concerning its function in muscle regeneration.
a) See answer to point 1
  b) Future studies by experts, hopefully using the panel of antibodies against actin isoforms, will provide more information for the participation of satellite cells during the process of muscle regeneration.
